# Thoracoabdominal asynchrony: Two methods in healthy, COPD, and interstitial lung disease patients

**DOI:** 10.1371/journal.pone.0182417

**Published:** 2017-08-02

**Authors:** Mayra Caleffi Pereira, Desiderio Cano Porras, Adriana Claudia Lunardi, Cibele Cristine Berto Marques da Silva, Renata Cléia Claudino Barbosa, Letícia Zumpano Cardenas, Renata Pletsch, Jeferson George Ferreira, Isac de Castro, Celso Ricardo Fernandes de Carvalho, Pedro Caruso, Carlos Roberto Ribeiro de Carvalho, André Luis Pereira de Albuquerque

**Affiliations:** 1 Pulmonary Division, Heart Institute (Incor), Hospital das Clínicas da Faculdade de Medicina da Universidade de São Paulo, São Paulo, Brazil; 2 Sírio-Libanês Teaching and Research Institute, São Paulo, Brazil; 3 Department of Physical Therapy, Hospital das Clínicas da Faculdade de Medicina da Universidade de São Paulo, São Paulo, Brazil; Telethon Institute for Child Health Research, AUSTRALIA

## Abstract

**Background:**

Thoracoabdominal asynchrony is the nonparallel motion of the ribcage and abdomen. It is estimated by using respiratory inductive plethysmography and, recently, using optoelectronic plethysmography; however the agreement of measurements between these 2 techniques is unknown. Therefore, the present study compared respiratory inductive plethysmography with optoelectronic plethysmography for measuring thoracoabdominal asynchrony to see if the measurements were similar or different.

**Methods:**

27 individuals (9 healthy subjects, 9 patients with interstitial lung disease, and 9 with chronic obstructive pulmonary disease performed 2 cycle ergometer tests with respiratory inductive plethysmography or optoelectronic plethysmography in a random order. Thoracoabdominal asynchrony was evaluated at rest, and at 50% and 75% of maximal workload between the superior ribcage and abdomen using a phase angle.

**Results:**

Thoracoabdominal asynchrony values were very similar in both approaches not only at rest but also with exercise, with no statistical difference. There was a good correlation between the methods and the Phase angle values were within the limits of agreement in the Bland-Altman analysis.

**Conclusion:**

Thoracoabdominal asynchrony measured by optoelectronic plethysmography and respiratory inductive plethysmography results in similar values and has a satisfactory agreement at rest and even for different exercise intensities in these groups.

## Introduction

Thoracoabdominal asynchrony (TAA) is the nonparallel motion or even the opposing movement of the rib cage (RC) and abdomen (AB) during inspiration [1,2]. The suspected mechanism of this asynchrony is the uncoordinated action of respiratory muscles, mainly inspiratory accessory muscles and the diaphragm, which are related to changes in lung mechanics [3]. This condition has been found in different clinical situations, such as in children with neuromuscular disorders [4] and even in intensive care units as a marker of failure of mechanical ventilation [5]. In chronic obstructive pulmonary disease (COPD), it is related to airflow obstruction and results in more breathlessness and early chest wall hyperinflation during exercise [6].

Respiratory inductive plethysmography (RIP) and optoelectronic plethysmography (OEP) are methods used to measure TAA. RIP uses the bicompartmental principle proposed by Konno and Mead [7], considering the 2-dimensional variation of RC and AB by measuring the cross-sectional area [8,9]. OEP is a tricompartmental model described by Ward et al. [10], in which the ribcage is not unique but composed of upper and lower regions, and provides the 3-dimensional analysis of upper rib cage (RCp), lower rib cage (RC_AB_), and AB [11–14]. Both methods have been widely used to evaluate TAA in recent years in several diseases, such as COPD [6,15–18], gastric disorders [19,20], and chest [21] postoperative surgery, spinal cord injury [22], and even in healthy subjects [23–26]. Although there is no study related to TAA in adults with interstitial lung disease (ILD), the altered respiratory mechanics affects the exercise performance in these patients, which again highlights the clinical relevance of the studies related to TAA [27]. Although OEP measures volume variation and allows evaluating the RC_AB_ more appropriately, it is more costly and is not available at many centers. Thus, it is of great importance to have a study comparing these 2 methods in different diseases.

The main objective of this study was to describe the agreement between TAA values obtained by RIP and OEP not only in healthy subjects, but also in patients with COPD and ILD at rest, and during moderate and intense exercise.

Our hypothesis is that in similar ventilatory conditions, both at rest and at different exercise intensities, the synchrony values between RCp and AB are similar when obtained by the 2 methods. This finding would help in the choice of which method to use, depending on the study outcomes and laboratory conditions.

## Material and methods

### Subjects

This was a cross-sectional study involving 27 consecutive individuals: 9 healthy subjects (FEV_1_ > 80% pred and FVC > 80% pred), 9 subjects with ILD (FVC < 80% pred), and 9 with COPD (FEV_1_ < 50% pred). The healthy subjects were composed by volunteers from the research group and individuals recruited in the Hospital, paired by age, BMI and gender. The patients were derived from the ambulatory COPD and ILD Respiratory Department at the Heart Institute (Incor), Hospital das Clínicas da Faculdade de Medicina da Universidade de São Paulo (HC-FMUSP). The study was approved by the local Ethics Committee (Committee for the Analysis of Research Projects (CAPPesq), of Incor/HC-FMUSP (protocol number: 3712/11/30), and all subjects signed the informed consent.

Were included the healthy subjects with normal lung function and physically inactive (exercise activity less than twice a week), and patients which had been clinically stable for at least the 2 previous months and did not require chronic oxygen supplementation. Those with non-pulmonary restrictive ventilatory disorder, such as muscular dystrophies or conformational chest wall changes (kyphosis), were not considered.

### Lung function tests

All measurements were performed according to American Thoracic Society/European Respiratory Society (ATS/ERS) guidelines [28–30]. Spirometry was performed using a calibrated pneumotachograph (Medical Graphics Corporation—MGC, St Paul, MN, USA), whereas lung volumes and carbon monoxide diffusion capacity (D_L_CO) were obtained on a body plethysmograph (Elite Dx, Elite Series^TM^–MGC). The following variables were obtained: forced vital capacity (FVC), expiratory forced volume in the first second (FEV_1_), TLC (total lung capacity), RV (residual volume), and carbon monoxide diffusion capacity (D_L_CO). The healthy subjects underwent only forced spirometry to measure FEV_1_ and FVC.

### Cardiopulmonary test

All the individuals previously had performed a maximal cardiopulmonary test (VMax, CareFusion, USA) limited by exhaustion on cycle ergometer (Corival, Lode B.V., Groningen, The Netherlands). The protocol consisted of 2 minutes of rest, 2 minutes of unloading followed by an increment 10–20 Watts, according to the physical level evaluated subjectively by the physician. Oxygen saturation (SpO_2_), measured by pulse oximetry (Onyx, model 9500; Nonin, Plymouth, MN, USA), and electrocardiography (CardioPerfect; Welch Allyn, Inc., Skaneateles Falls, NY, USA) were monitored continuously during the test. Afterwards, the subjects performed the submaximal tests with OEP and RIP, with an interval more than 24 hours between the maximal and the submaximal tests.

**Fig 1:**
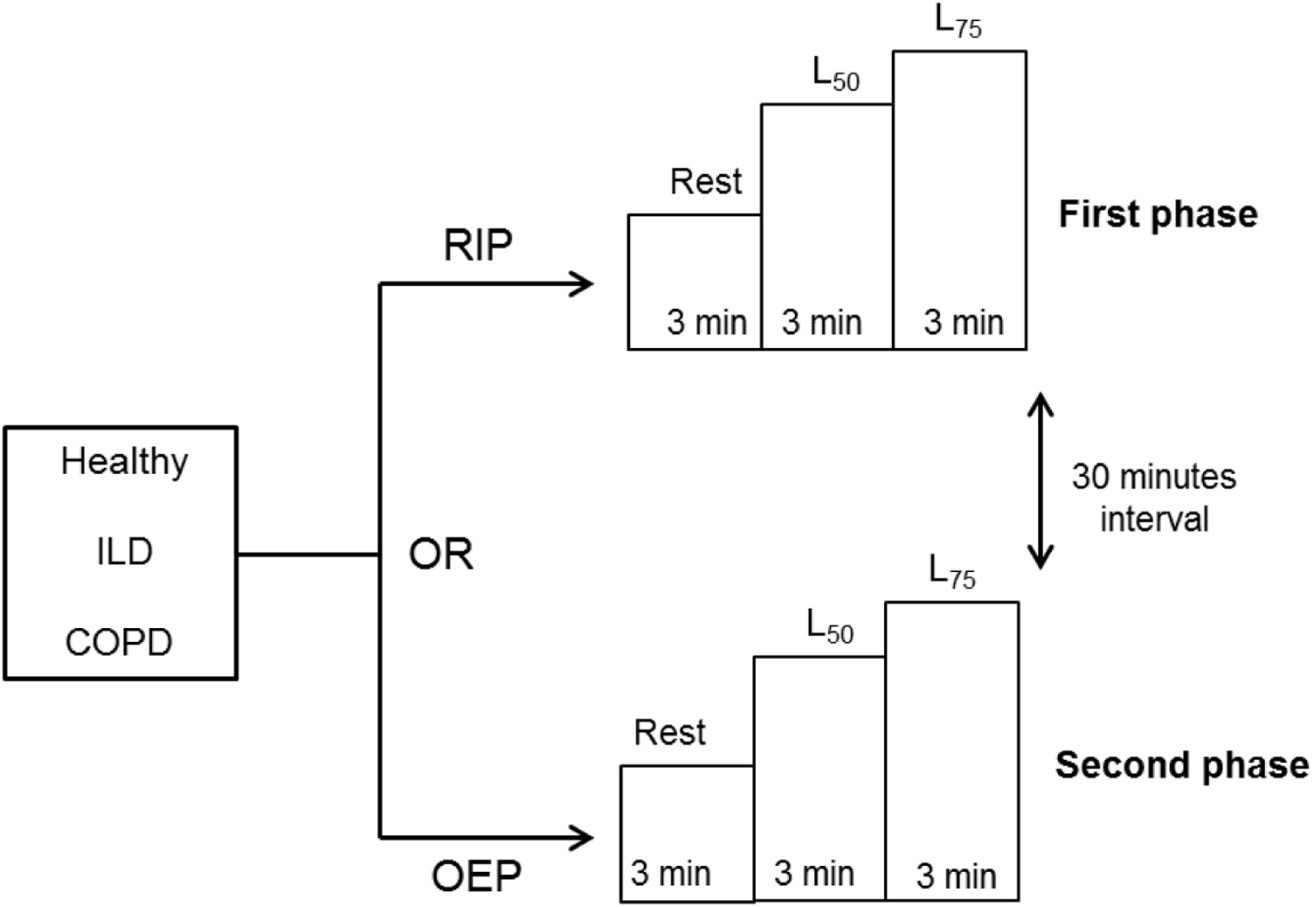
Study protocol. **Monitoring in the first and second phases, depending on the randomization.** OEP: optoelectronic plethysmography, RIP: respiratory inductive plethysmography, L50 and L75: 50% and 75% of maximal load.

The order of measurements of TAA was randomized, and the protocol consisted of 2 phases (Fig 1). First, the subject remained at rest for 3 minutes followed by submaximal exercise on a cycle ergometer, including 3 minutes at 50% (L_50_) and 3 minutes at 75% (L_75_) of maximal load obtained previously in the cardiopulmonary test. In the second phase, after a 30-minute interval, the same sequence was repeated with subsequent monitoring. The individuals maintained a seated position at all times on the cycle ergometer with flexed and abducted arms supported laterally by a stick to avoid interference in data measurement. This position is mandatory for good acquisition with OEP, and it was maintained during RIP acquisition to simulate the same body posture and muscle recruitments.

At the beginning and the end of each moment, the individuals were asked to score dyspnea and leg effort with the Borg Scale of Perceived Exertion [31]. During the protocol, the subjects were monitored with a pneumotachograph (Pneumotach flow-head 3830-Hans Rudolph Inc, USA) coupled with an interface (7900 Series Mouth Breathing Only Face Mask, Hans Rudolph Inc, USA) continuously at rest and during exercise to measure the respiratory rate (RR), tidal volume (VT), and ventilation (VE). It was calibrated with a 3-liter syringe (Hans Rudolph Inc, USA) as standardized by Miller et al. [29].

### Optoelectronic plethysmography (OEP system, BTS, Milan, Italy)

**Fig 2:**
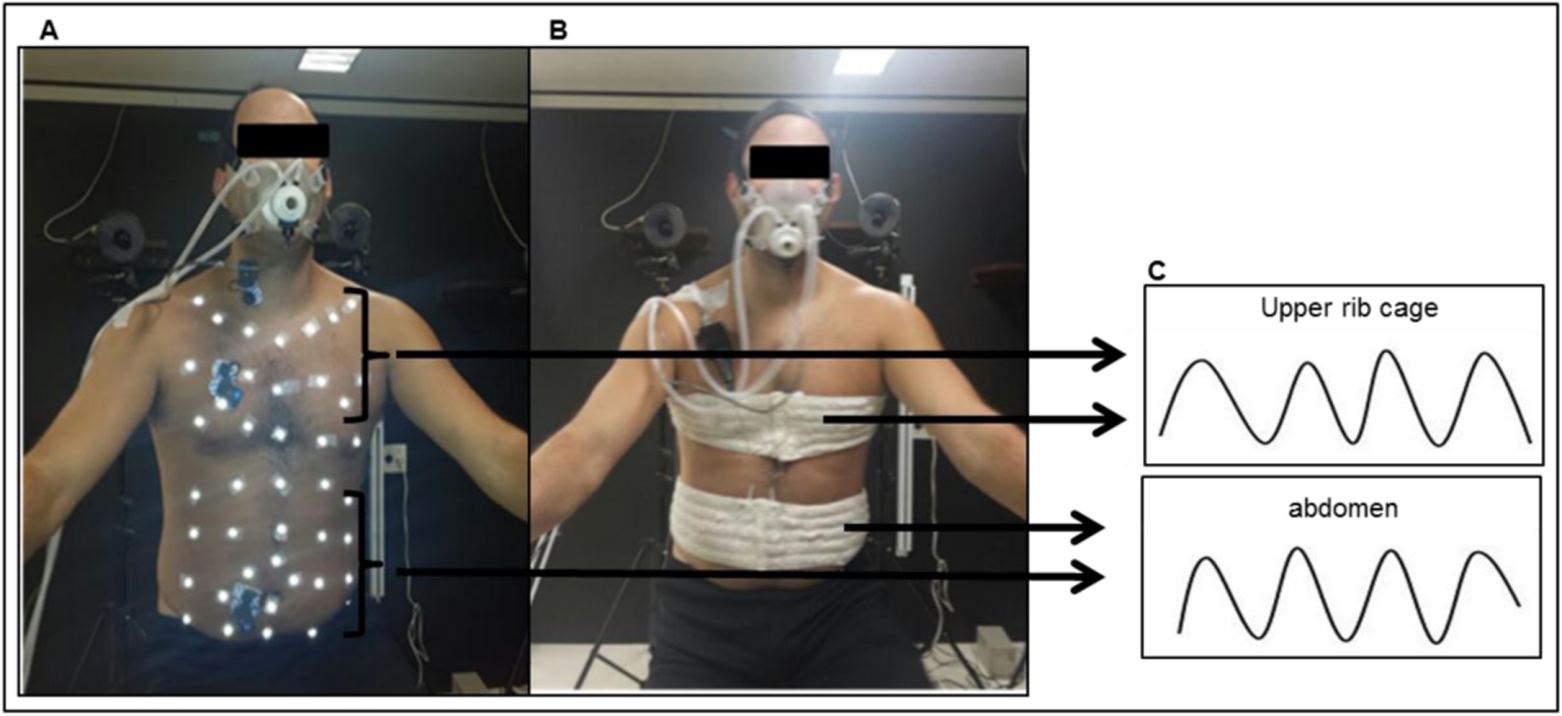
Monitoring. A) Optoelectronic plethysmography (OEP): retro-reflective markers separating both the upper rib cage (RCp) and abdomen (AB) compartments. B) Respiratory Inductive plethysmography (RIP): elastic bands positioned on RCp and AB compartments. C) RCp and AB movement during respiratory cicles.

Kinematics of the chest wall by OEP was measured by 89 retro-reflective markers attached to the trunk, on precise anatomic points to separate the 3 compartments: RCp, RCab, and AB, as described previously by Aliverti et al. [6] (Fig 2A). Eight TV cameras (4 in front of and 4 in back of the subject) operating at 100 frames per second captured the position of the markers, creating a 3-dimensional image of the compartments. Three-dimensional calibration of the equipment was performed as recommended by the manufacturer. Because only the RCp and AB were evaluated in respect to synchrony during inspiration, the volume of RCab was not considered.

### Respiratory inductive plethysmography (Respitrace®, Nims, Miami, FL, USA)

The system is composed by 2 bands (Teflon®-coated inductance bands) placed on the RC and AB to measure the changes in the cross-sectional area of these compartments during respiration. The RC band was placed at the axillar level and the AB band at the umbilical level (Fig 2B). RC and AB channels were calibrated to obtain their electrical gains using a specific procedure [32].

### Data analysis

**Fig 3:**
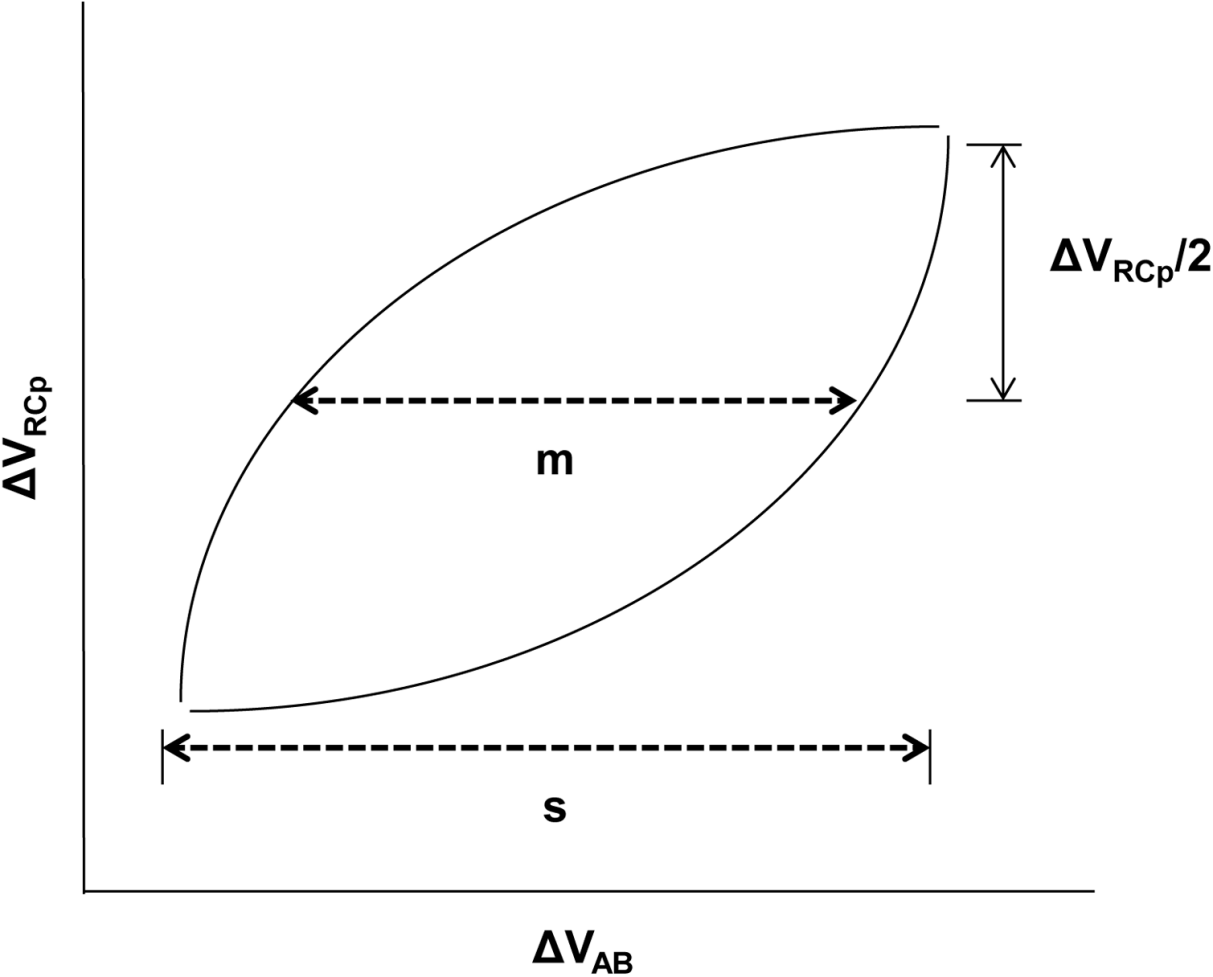
Representation of the PhAng calculation. ΔV_RCp_: upper RC variation; ΔV_AB_: AB abdomen variation, m: 50% of ΔV_RCp_, s: maximal excursion of ΔV_AB_. PhAng: Sin Ɵ = m/s.

One average cycle was obtained derived from 5 stable respiratory cycles in the last minute at each intensity (rest, L_50_, and L_75_). From this average cycle, the inspiratory synchrony was measured using the Lissajous approach in which the upper RC variation (Δ_RCp_) is plotted against AB variation (Δ_AB_), where “m” represents the line parallel to the x-axis at 50% of Δ_RCp_, “s” is the maximal excursion of AB Δ_AB_, and PhAng is defined by Sin Ɵ = m/s (Fig 3), as the approach used previously by Aliverti et al. [6]. This principle was used in the OEP and RIP analysis. The PhAng values range from 0° (perfect synchrony) to 180° (paradoxal movement), and positive angles means that RCp excursion is ahead of AB excursion, while negative angles mean the opposite [17]. The same average respiratory cycles were used to analyze the ventilatory variables.

### Statistical analysis

This study analyzed the agreement degree between methods by using an agreement index between the 2 methods of ≥ 0.7 (linearity of the model) for the null hypothesis (H0) rejection and an index of agreement ≤ 0.4 for H0 acceptance. A risk of α ≤ 5% (0.05) was considered for type I or first species error and β ≤ 20% for type II or second species error. A sample size of 58 evaluations for a power of 90% was estimated.

The Shapiro-Wilk test was used to test the normality of data distribution. For the comparison of the 3 groups, the ANOVA test was performed followed by Tukey’s post hoc test. The paired *t* test was used to compare pulmonary function variables between COPD versus ILD groups, and for respiratory variables and PhAng obtained by OEP versus RIP monitoring. A regression polynomial model (quadratic) was used to analyze the correlation, and Bland-Altman technique [33] to analyze the agreement between the methods. The level of significance was set at 0.05.

Statistical analysis was performed using SPSS 21.0 software (IBM SPSS Statistics®, US).

## Results

Table 1 shows the demographic, anthropometric, and lung function data of the 3 groups. There were no significant differences between groups in sex, age, and body mass index (BMI). Healthy subjects had no comorbidity, whereas the COPD group had 2 patients with previous coronary insufficiency and peripheral vascular disease. Former smokers were more prevalent in COPDs, with just 22% in ILD. Four patients had hypersensitivity pneumonitis, 1 had idiopathic pulmonary fibrosis, 1 had nonspecific interstitial pneumonia, and 3 had rheumatologic disease as the cause of ILD. The patients with rheumatologic causes did not have musculoskeletal impairment that could interfere with the performance of the protocol.

**Table 1:** Demographic and pulmonary function characteristics of healthy, ILD, and COPD groups.

	Healthy (n = 9)	ILD (n = 9)	COPD (n = 9)
**Demographic/Anthropometric**			
Male (%)	5 (56)	5 (56)	6 (67)
Age, yrs	50 ± 5.5	50 ± 13.1	60 ± 4.4
BMI, kg/m^2^	26.2 ± 1.7	26 ± 2.8	24 ± 5.1
**Comorbidities**			
Systemic arterial hypertension (%)	-	1 (11)	2 (22)
Coronary insufficiency (%)	-	-	1 (11)
Peripheral vascular disease (%)	-	-	1 (11)
Previous Smoking (%)	-	2 (22)	7 (78)
Pack years	-	34 ± 5.6	70 ± 62.4
**Pulmonary function**			
FEV_1_ L	2.9 ± 0.4	1.7 ± 0.5*	1.1 ± 0.3*^,^ ^a^
%predicted	95.8 ± 11.7	57 ± 9.2*	38 ± 12.6*^,^ ^a^
FVC L	3.7 ± 0.6	2.1 ± 0.7*	2.8 ± 0.6*
% predicted	96.4 ± 14.2	57.2 ± 10.8*	73.8 ± 11.8*
FEV_1_/FVC	0.8 ± 4.5	0.82 ± 6.5	0.41 ± 10.5*^,^ ^a^
% predicted	100 ± 6.4	100 ± 7.6	50.2 ± 11.8*^,^ ^a^
TLC L	-	4.2 ± 0.9	7.3 ± 1.3 ^a^
% predicted	-	74.8 ± 12.5	124.4 ± 19.9 ^a^
RV L	-	1.9 ± 0.9	3.8 ± 1.3 ^a^
% predicted	-	85.6 ± 8.4	189.4 ± 67.9 ^a^
DL_CO_ ml/mmHg/min	-	11.3±3.9	11.6±7.1
% predicted	-	47.7 ± 9.2	44 ± 27.5
IC L	2.4 ± 0.4	1.2 ± 0.4	2.1 ± 0.5
% predicted	79.5 ± 13.1	41.1 ± 13.5	69.4 ± 15.1

ILD: interstitial lung disease, COPD: chronic obstructive pulmonary disease, BMI: body mass index, FEV_1_: forced expiratory volume in 1 second, FVC: forced vital capacity, TPC: total lung capacity, RV: residual volume, DL_CO_: carbon monoxide diffusion capacity, IC: inspiratory capacity. Data presented as average ± SD or n (%).

* p < 0.05 compared with healthy and

^a^ p < 0.05 compared with ILD.

The COPD group was characterized by severe airflow obstruction with FEV1 <50% of predicted, experiencing also lung hyperinflation, increased RV and decreased DLco. Patients with ILD were functionally impaired as well, with reduced FVC and DLco (Table 1).

No significant differences were found in the different intensities (rest, L_50_, and L_75_), between RR, VT, and VE obtained with OEP and RIP, except for VE in COPD and ILD groups at L_50_ and L_75_ intensities (Table 2), but without a difference in VT and RR at these moments. Also, the Borg scale scores were not significantly different when applied at the first and second phase (S1 Table).

**Table 2:** Ventilatory variables of healthy, ILD, and COPD groups at rest, L_50_, and L_75_ during OEP and RIP monitoring.

		RR (per minute)		VT (L)		VE (L/min)	
Groups		OEP	RIP	OEP	RIP	OEP	RIP
**Healthy (n = 9)**	**Rest**	17.6 ± 5.2	18.8 ± 3.5	0.6 ± 0.4	0.6 ± 0.3	9.8 ± 4.3	10.8 ± 4.6
	**L**_**50**_	21.3 ± 5.4	21.1 ± 4.3	1.2 ± 0.6	1 ± 0.4	23.6 ± 12.2	18.7 ± 11.2
	**L**_**75**_	24.5 ± 5.8	24.2 ± 5.1	1.5 ± 0.8	1.2 ± 0.6	36.7 ± 20.5	29 ± 19.2
**ILD** **(n = 9)**	**Rest**	22.7 ± 5.2	23.5 ± 6.5	0.5 ± 0.1	0.5 ± 0.2	11.7 ± 2,7	11.4 ± 2.4
	**L**_**50**_	29 ± 6.4	27.1 ± 7.4	0.8 ± 0.2	0.7 ± 0.2	22.7 ± 3.1*	18.4 ± 4
	**L**_**75**_	33.3 ± 7.3	31.2 ± 8.5	1 ± 0.3	0.9 ± 0.3	30.5 ± 6*	25.8 ± 6.2
**COPD (n = 9)**	**Rest**	20.7 ± 3.3	19 ± 3.3	0.7 ± 0.2	0.7 ± 0.2	13 ± 2,7	12.8 ± 3
	**L**_**50**_	21.7 ± 3.4	20.3 ± 3	0.9 ± 0.2	0.8 ± 0.2	18.7 ± 4,8*	16.7 ± 3.4
	**L**_**75**_	23.4 ± 4	21.7 ± 3.3	1 ± 0.2	1 ± 0.2	23.6 ± 6*	20.8 ± 5.2

ILD: interstitial lung disease, COPD: chronic obstructive pulmonary disease, OEP: optoelectronic plethysmography, RIP: respiratory inductive plethysmography, RR: respiratory rate, VT: tidal volume, VE: ventilation. Data presented as mean ± SD.

*p<0.05 OEP *vs* RIP.

Two COPD patients had peripheral oxygen saturation (SpO2) < 90% during exercise, so they were supplemented with oxygen to ensure SpO2 > 90%.

**Fig 4:**
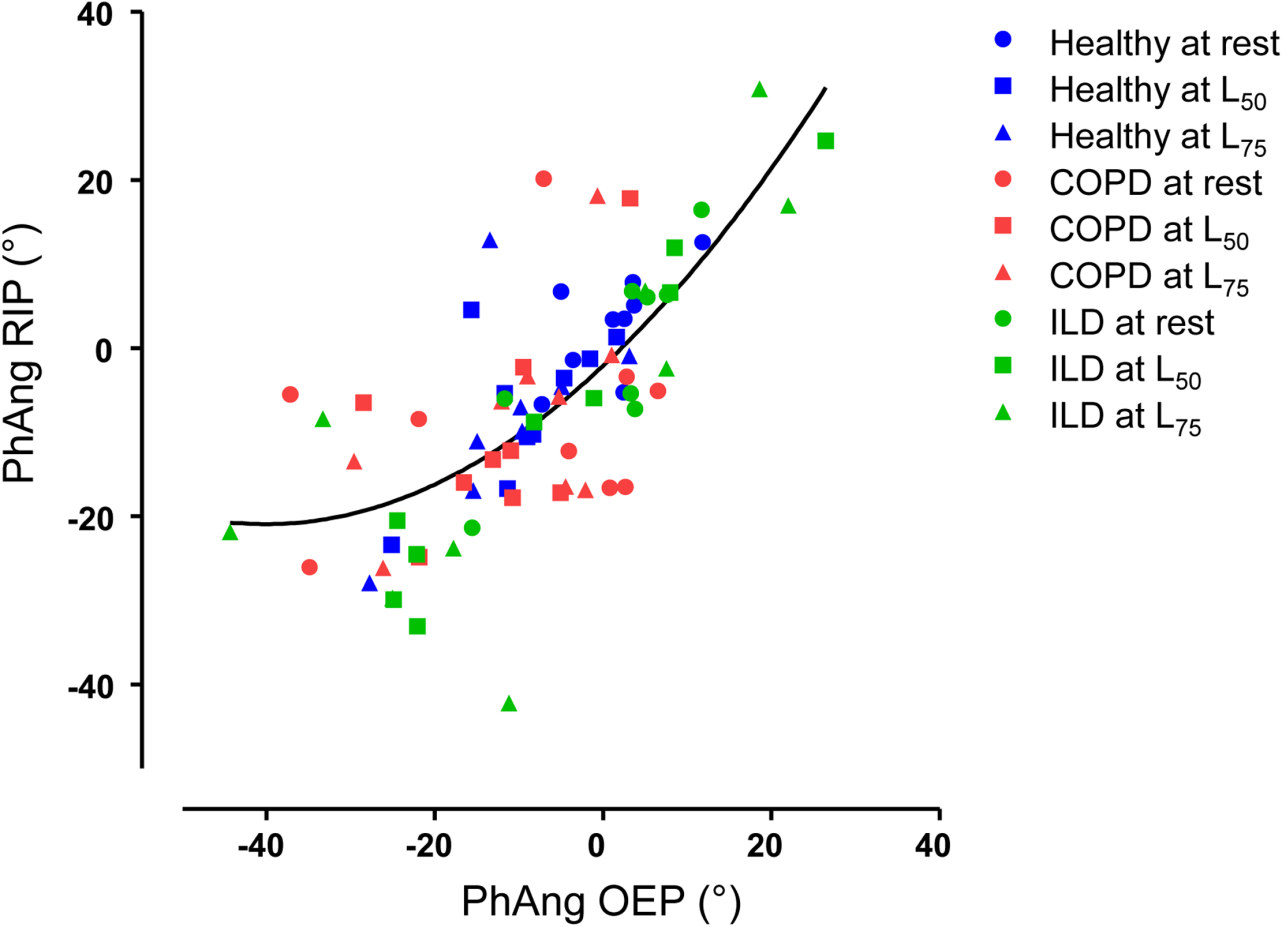
Regression polynomial model representing the PhAng with OEP and RIP. PhAng: Phase Angle, OEP: optoelectronic plethysmography, RIP: respiratory inductive plethysmography, ILD: interstitial lung disease, COPD: chronic obstructive pulmonary disease, L50 and L75: 50% and 75% of maximal load.

**Fig 5:**
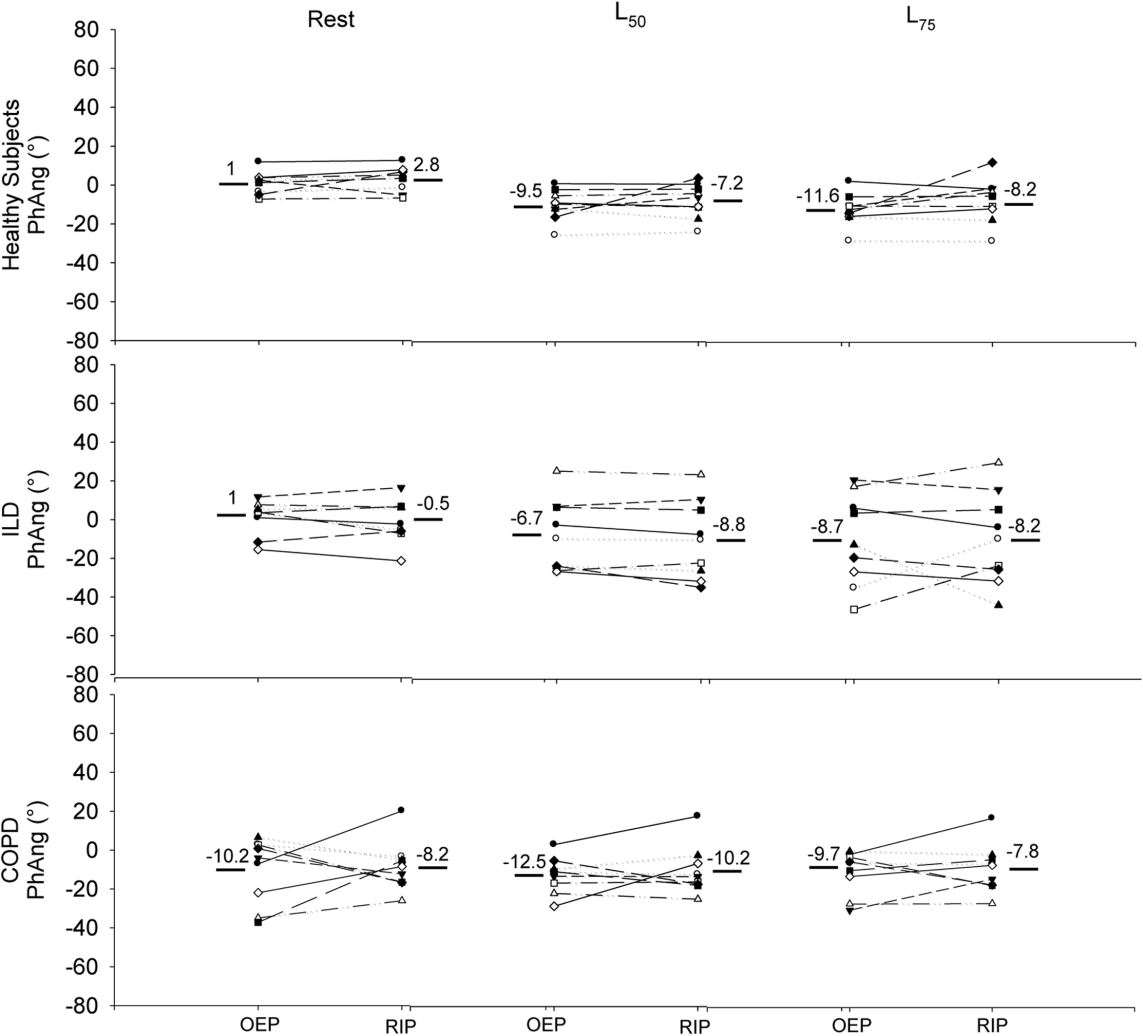
PhAng obtained with both methods in healthy, ILD, and COPD at rest and during exercise. PhAng: Phase Angle, OEP: optoelectronic plethysmography, RIP: respiratory inductive plethysmography, ILD: interstitial lung disease, COPD: chronic obstructive pulmonary disease, L50 and L75: 50% and 75% of maximal load.

Fig 4 shows the PhAng values obtained with OEP and RIP at different intensities in all individuals. Using a regression polynomial model (quadratic), we obtained r^2^ = 0.53 or r = 0.73 (p< 0.0001), without outliers. Analyzing the groups separately (Fig 5), in healthy, the individual values of PhAng were very similar at rest and even during exercise (L_50_ and L_75_), with little variability between them comparing the methods. For the ILD group, draw attention the higher range of PhAng values with exercise progression, regardless of OEP or RIP measurement. At intense exercise (L_75_), 3 patients with ILD increased the difference between methods. The COPD group had high dispersion of PhAng values and a higher difference between methods at rest. Of interest, in the effort (L_50_ and L_75_), there was less variability of the PhAng values and less difference between methods for each subject. There was no statistical difference between the methods neither for the different exercise intensities nor for the groups.

**Fig 6:**
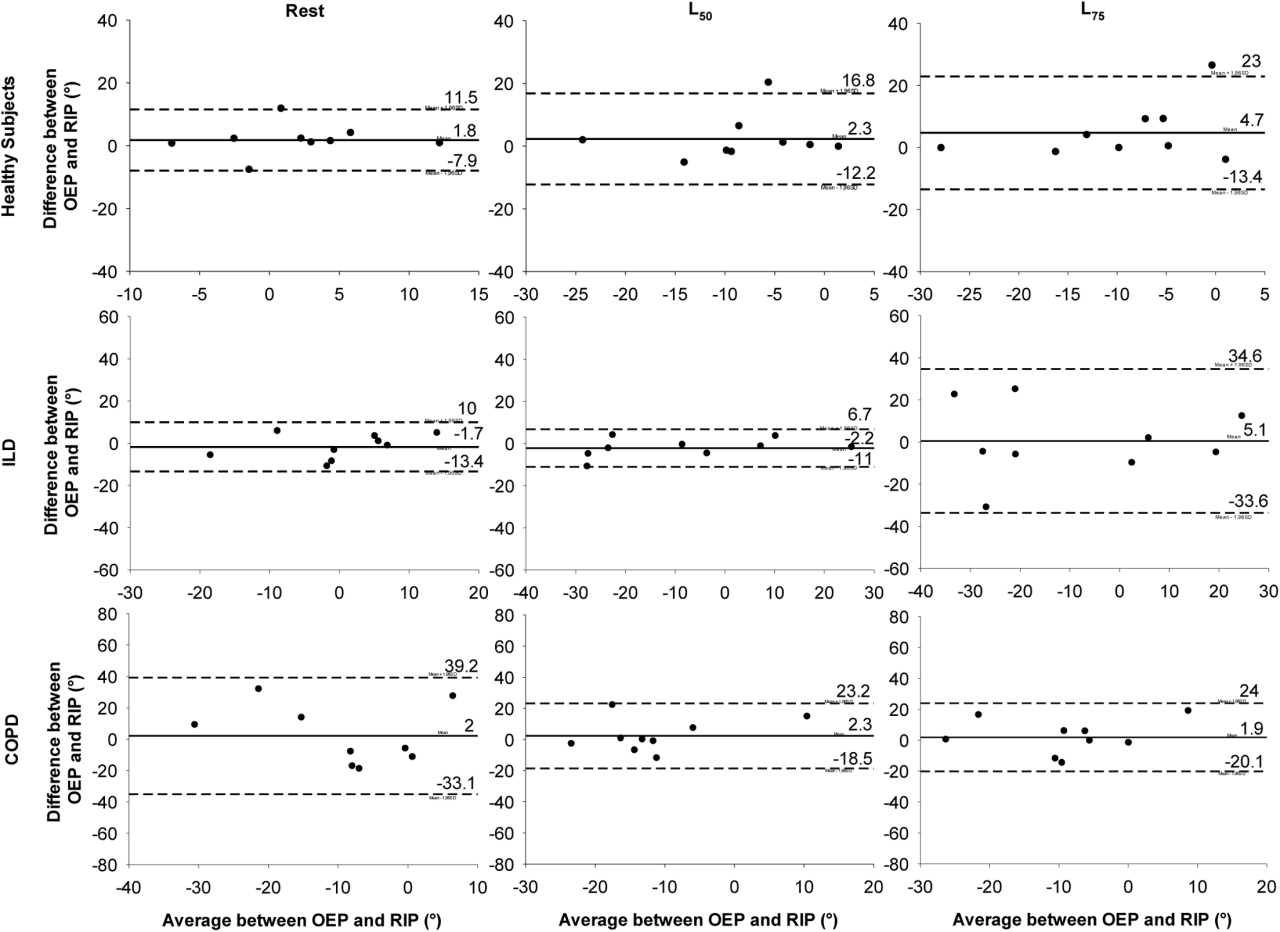
Bland-Altman graph representing the agreement between OEP and RIP. OEP: optoelectronic plethysmography, RIP: respiratory inductive plethysmography, ILD: interstitial lung disease, COPD: chronic obstructive pulmonary disease, L50 and L75: 50% and 75% of maximal load. Solid line: mean difference between the 2 methods, Dotted lines: upper and lower limit of agreement (mean ± 1.96 SD).

Regarding agreement between the OEP and RIP methods for measuring TAA, the Bland & Altman analysis (Fig 6) shows that during rest the mean difference between OEP and RIP was near zero with low dispersion between the healthy and ILD groups. For the COPD group, although the mean difference was close to zero, the difference between RIP and OEP was greater. During exercise, in healthy subjects the mean difference remained similar at L_50_, with a small increase in dispersion of this difference at L_75_. In patients with ILD, only at L_75_ was there an increase in the dispersion. Finally, for COPD at both L_50_ and L_75_ intensities, the mean difference remained low and a decrease occurred in the dispersion.

## Discussion

The evaluation of PhAng values is of great importance, since it is a measure widely used to access TAA [6, 15, 17, 19–25]. Asynchrony between respiratory compartments has been described in different diseases, as neuromuscular disorders [4, 22], related to mechanical ventilation [5], COPD [6, 15–18] and post-operative [19, 20], with clinical repercussions as higher dyspnoea, early dynamic hyperinflation and failure of mechanical ventilation related to these diseases. Also, thoracoabdominal motion is described in healthy subjects [25, 26]. In this context, the study of TAA is clinically relevant. Moreover, we considered the TAA evaluation during exercise (mainly moderate and intense levels) important, since this finding may not be found at rest.

It is strictly relevant to clarify which method can be used for this evaluation. We found that in general OEP and RIP results were in agreement when measuring TAA. In healthy individuals, both methods resulted in similar values of PhAng and small differences at rest and during exercise. In ILD, just at L_75_ the difference between methods was high. Finally, in patients with severe COPD the difference between OEP and RIP was reduced during exercise and increased only at rest, with a higher dispersion of individual PhAng values. Although the variation in PhAng values between the methods in some individuals, as at L_75_ in ILD and at rest in COPD, there was no statistical difference.

OEP has been the method used most in recent years to evaluate the asynchrony between compartments, because it is possible to measure the volume variation; however, RIP is used by many centers. Previous studies with RIP involving healthy subjects and those with COPD have shown the influence of age and mainly exercise intensities on PhAng [18,24]. This influence on the asynchrony between thoracic and abdominal compartments was also found when using OEP with exercise modifying the TAA in COPDs [6]. However, it is notable that both OEP and RIP have different principles of measurement and important differences, such as the cost, ease of use, and even the amount of space available in the lab.

Although most studies evaluated healthy subjects and those with COPD, we decided to also investigate ILD patients, because they have an augmented respiratory workload and an alteration in breathing pattern with more breathlessness [27]. In addition, our individuals were evaluated not only at rest, but also at moderate effort (L_50_) and at more intense (L_75_) levels of exercise.

The PhAng values obtained in healthy subjects, regardless of method, were very similar at rest and at both exercise intensities. There was an increase in TAA, but this behavior was similar in the 2 methods. Also, there was a pattern of negative angles, probably due to early recruitment of abdominal muscles, related to chest wall expansion during exercise.

It is known that, in patients with ILD, exercise performance is affected by several factors, including respiratory mechanics, with low tidal volume and high respiratory rates [27]. Nevertheless, we did not find published studies about PhAng in adults with ILD. In our results, we noted a large variability in the PhAng values during both exercise intensities (L_50_ and L_75_). The correlation between these methods was high, and the difference between them was increased only at L_75,_ the level at which the range of individual PhAng was greater no matter whether OEP or RIP. One possible reason for this higher difference at L_75_ is the greater ventilation with more shallow breathing in OEP, because the pattern of breathing interferes with the recruitment of chest wall compartments [12]. Note that in ILD, the range of PhAng increases more intensively than in COPD during exercise, which happened with ventilation and respiratory rate primarily.

In the COPD group, the greater difference between the 2 methods was found at rest, where the range of PhAng was high in both methods as well. On the contrary, less variability occurred in the PhAng values during exercise (L_50_ and L_75_). Because our study evaluated only patients with severe COPD, this significant range of PhAng and breathing pattern is expected even at rest. Regardless of the method, the PhAng values were negative at rest and exercise. A previous study using RIP in COPD also found a greater contribution from the abdomen during exercise progression [18]. Taking this into account, we suggest that TAA in COPD depends on multiple factors, such as the degree of activation of the inspiratory (accessory and diaphragm) and expiratory muscles [6,16] and the pattern of breathing.

A previous study [6] shows that the highest degree of TAA between RC_AB_ and AB was in patients who had dynamic hyperinflation, while another [15] did not find this result, which makes the relationship between dynamic hyperinflation and TAA in COPD still controversial. During maximal exercise, our patients experienced some degree of hyperinflation (ΔIC -22.4±16.4% baseline); however, we did not obtain such a measure during the exercise to confirm whether there was an influence on TAA.

Our study has some limitations, such as the inability to perform the acquisition using both methods simultaneously. Certainly this was the major limitation, although we made sure the individuals were under similar ventilatory conditions in the 2 phases. These results cannot be extrapolated to the asynchrony between RCp and RC_AB_ compartments, because it was only evaluated in RCp and AB. Although some studies have found asynchrony mainly between ribcage compartments [6,17], we consider that RIP is not sensitive enough to measure the small changes in RC_AB_, and it can be even more difficult in women because of the breasts.

## Conclusions

This is the first study comparing these 2 methods in 3 different populations, in which we found that both OEP and RIP were concordant for obtaining PhAng values under most of the conditions tested, mainly in healthy individuals. For severe COPD and ILD, both methods had good agreement, except when there was a wide variability in PhAng. In addition, the 2 methods reflected the same PhAng behavior, considering the responses from exercise compared to rest. Therefore, this study can contribute to the decision about which is the best method for evaluating thoracoabdominal asynchrony, considering the advantages and disadvantages of each one, and other factors, such as study design, purpose, and the physical structure of the laboratory.

## Supporting information

S1 Table Borg scale score applied at the beginning and at the end of each phase of the protocol. ILD: interstitial lung disease, COPD: chronic obstructive pulmonary disease. Data presented as mean (max-min).
(PDF)
