# Supplementary material for: Thoracoabdominal asynchrony: Two methods in healthy, COPD, and interstitial lung disease patients
Source: PLoS One. 2017 Aug 2;12(8):e0182417. doi: 10.1371/journal.pone.0182417 (PMC5540557; doi:10.1371/journal.pone.0182417)
Supplement: S1 Table — ILD: interstitial lung disease, COPD: chronic obstructive pulmonary disease. Data presented as mean (max-min). (PDF) [file pone.0182417.s001.pdf]

S1 Table. Borg Scale score applied at the beginning and at the end of each phase of the protocol.

Groups		Dyspnea		Legs	
	Borg	Initial	End	Initial	End
**Healthy (n=9)**	1° phase	0 (0-0)	2,7 (0,5-5)	0 (0-0)	3,3 (0,5-7)
	2° phase	0,7 (0-3)	3,8 (1-8)	0,8 (0-3)	3,8 (0,5-7)
**ILD (n=9)**	1° phase	0,7 (0-2)	3,7 (0-9)	1,2 (0-5)	4,3 (0-9)
	2° phase	1,3 (0-4)	4 (0,5-10)	1,9 (0-6)	4,7 (0-10)
**COPD (n=9)**	1° phase	0,5 (0-2)	3 (0,5-5)	0,7 (0-2)	3 (0,5-5)
	2° phase	0,4 (0-1)	3,2 (0,5-5)	0,4 (0-1)	3 (0,5-5)

ILD: interstitial lung disease, COPD: chronic obstructive pulmonary disease. Data presented as mean (max-min).
